# A rare clinical image of fourth-degree bedsore

**DOI:** 10.11604/pamj.2022.42.271.36133

**Published:** 2022-08-11

**Authors:** Minal Dambhare, Deeplata Mendhe

**Affiliations:** 1 Nursing Tutor Florence Nightingale Training College of Nursing, Datta Meghe Institute of Medical Science (DU) Sawangi Meghe Wardha, Wardha, Maharashtra, India,; 2 Department of Community Health Nursing, Smt. Radhikabai Meghe Memorial College of Nursing, Datta Meghe Institute of Medical Sciences (Deemed to be University), Sawangi, Wardha, Maharashtra, India

**Keywords:** Decubitus ulcer, spinal injury, bedsore

## Image in clinical medicine

Stage four bedsores are the most severe form of bedsores, also called pressure sores, pressure ulcers, or decubitus ulcers. A stage four bedsore is a deep wound reaching muscles, ligaments, or bones. They often cause residents to suffer extreme pain, infection, and invasive surgeries. Its major etiological factors are poor circulation, Immobility due to spinal cord injury, Excessive moisture, skin irritants like urine and feces, and friction. It mainly occurs in men and women, both aged 20 to 80 years. With early diagnosis and treatment, further complications can be prevented. We report a case of the 29-year-old patient; who came to the surgical intensive care unit with the complaints of swelling over the buttocks, in size of 20 x 20 x 8 cm bedsore covering both buttocks, foul-smelling he was apparently all right five years back when he noticed swelling and skin off on buttocks with itching and burning pain which was the insidious onset and progressive. Initially, the size was 20 x 20 x 8 cm, gradually increasing to the current size of 24 x 24 cm. He also complains of pain over the buttock, a throbbing type of pain. A blood investigation and skin biopsy were done, and the patient was referred to the surgical intensive care unit for further management.

**Figure 1:**
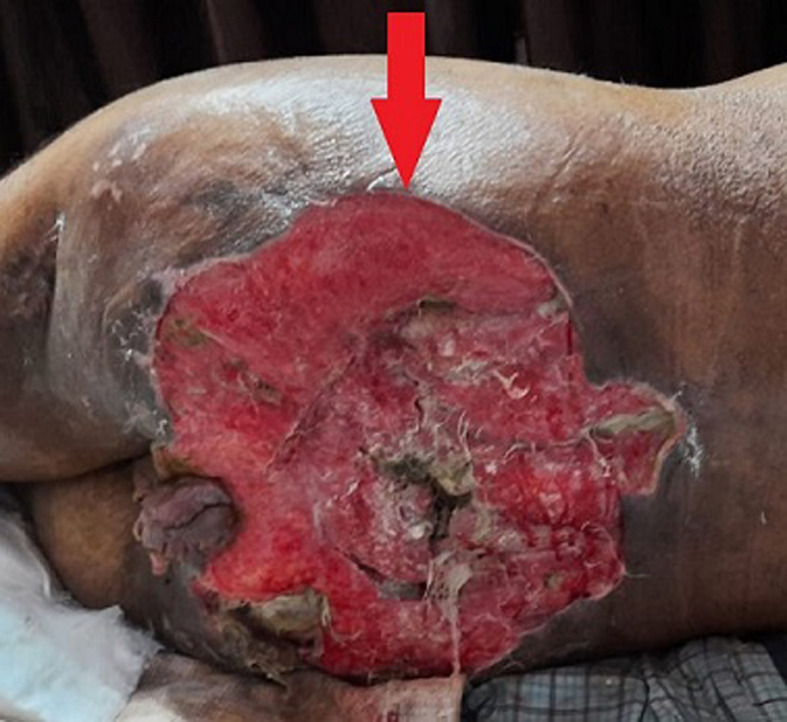
deep wound with redness of the skin

